# Characterization of biocarbon generated by high- and low-temperature pyrolysis of soy hulls and coffee chaff: for polymer composite applications

**DOI:** 10.1098/rsos.171970

**Published:** 2018-08-22

**Authors:** Peter Quosai, Andrew Anstey, Amar K. Mohanty, Manjusri Misra

**Affiliations:** 1 Bioproduct Development and Discovery Centre, Department of Plant Agriculture, University of Guelph, Guelph, Canada; 2 School of Engineering, University of Guelph, Guelph, Canada

**Keywords:** biocarbon, coffee chaff, pyrolysis, soy hulls

## Abstract

The physical properties of biocarbon vary widely with the biomass used, and the temperature and duration of pyrolysis. This study identifies the effects of feedstock characteristics and pyrolysis conditions on the production of biocarbon and the corresponding properties for industrial applications. For coffee chaff and soy hulls, ash content and carbon content increased with pyrolysis temperature and duration. Ash content increased thermal conductivity and specific heat, and decreased electrical conductivity. Change in surface area with pyrolysis conditions was dependent on type of feedstock. Increased surface area corresponded with increased thermal and electrical conductivity. Increased carbon content corresponded with increased graphitization and thermal stability and decreased surface functionality. Properties of soy hull biocarbons were found to be similar to the properties of other biocarbons with industrial applications such as incorporation into polymer composites.

## 1. Introduction

Coffee and soya beans are major agricultural products with 9 million metric tons of green coffee beans and 313 million metric tons of soya beans produced in 2016 worldwide [1,2]. The hulls or chaff from these products are low-value agricultural wastes which go to landfill when an agricultural use does not exist [3,4]. A promising use of this potential waste product is in the generation of biocarbon for composite applications as a bio-based replacement for mineral fillers such as glass fibre and talc [5,6]. Biocarbon has additional applications in carbon sequestration, as a soil amendment, in greenhouse growth media, filtration or as a precursor to activated carbon [7]. In these applications, this carbon-rich solid product is conventionally referred to as ‘biochar’; however, when it is tailored for value-added materials applications, it is referred to as biocarbon. Biocarbon is created as a primary product of slow pyrolysis of biomass and a co-product of bio-oil production by fast pyrolysis [8]. Pyrolysis is the process of heating a material, such as biomass, at temperatures between 400 and 900°C in the absence of oxygen to decompose the cellulose, hemicellulose and lignin of the biomass to a porous char, ash, bio-oil and syngas [8]. The products' relative proportions and physical properties vary widely with the biomass used, the temperature and duration of pyrolysis [9].

Fundamental research must be done to characterize new biocarbons from novel carbon sources, such as waste from agricultural industries [8,10,11]. Many biocarbon feedstocks have been characterized by previous studies, and will be used for comparison of material properties. Previously characterized biocarbons include miscanthus, switchgrass, poplar, apple and oak wood chips, rice husks, rice straw, corn stover, and purified lignin, cellulose and hemicellulose in varying ratios [8,9,12–14]. While biocarbon can be created alongside energy in a biomass power generation system, to produce a consistent product, a feedstock of regular quality and availability is essential. Consistent industrial wastes such as low-cost biomass are required as a feedstock for biocarbon production in order to use that biocarbon as a mainstream industrial product.

In the production of soy meal and soy oil from soya beans, the seed coat, or hulls, must be removed. These hulls are a low-value agricultural product which can be used as an additive to animal feed. However, the transport and storage cost of this product make pyrolysis for energy generation an economically viable option [15]. Owing to their availability, low cost and lack of competing applications, this study will investigate whether soy hulls can provide a consistent biomass feedstock to create a consistent biocarbon with desirable properties.

In the process of roasting coffee beans, the seed coats, also referred to as chaff, are removed as a waste product [16]. The temperature and duration of this roasting process is controlled to create lighter or darker coffee, light roast conditions could be as low as 200°C for 10 min, while dark roast conditions might be 320°C for 2 min [17]. The high disposal costs, including transport and landfill costs, and the consistency of coffee chaff make it another suitable candidate for consistent biocarbon production as a mainstream industrial product [4,16].

An important characteristic of biocarbon for industrial applications is its surface functionality, which affects the hygroscopic property of the carbon, the adsorption of gases and pollutants [18] and the adhesion of polymer composites [6]. The thermal stability of filler materials is an important consideration for polymer composite systems. The stability of the filler is a major factor in determining the processing window for a composite system. Natural fibres are typically incompatible with engineering polymers such as polyamides, as they thermally decompose at the high processing temperatures required for these polymers [19]. The thermal stability of biocarbon is advantageous because it can be extruded at higher temperatures than natural fibres. The electrical conductivity of biocarbon is also important as it affects the antistatic properties of composites made using the biocarbon. Conductivity is also a useful property in making valuable activated carbon, carbon electrodes and bio-based replacements for carbon black. The level of graphitization and carbonization affect the stability, density and functionality of biocarbon and can be determined through elemental or spectroscopic analyses [9]. This study aims to fully characterize these aspects in biocarbons obtained in a variety of processing conditions to determine their suitability for composite applications.

Understanding and controlling aspects such as surface chemistry, graphitization and conductivity are crucial for predicting how these materials will perform as reinforcing fillers or functional particles in a composite system. Ogunsona *et al*. [20] demonstrated that biocarbon produced at 500°C has considerably more surface functionality than biocarbon produced at 900°C, which resulted in superior interaction with a polyamide 6 (PA6) matrix. Their composites of PA6 with 500°C biocarbon had enhanced tensile and flexural strength due to the good interfacial adhesion between the polymer and biocarbon. From this study, it is clear that for a polar polymer such as PA6, less extreme pyrolysis conditions can be favourable in order to preserve some hydroxyl and carbonyl surface functionality in the biocarbon, improving the interaction with the matrix. A similar study by Behazin *et al*. [21] showed that biocarbon produced at 900°C greatly enhanced the modulus of polypropylene (PP)-biocarbon composites, while composites with 500°C biocarbon showed little improvement. Owing to the higher degree of graphitization provided by high-temperature pyrolysis, the 900°C biocarbon had a higher modulus and thus provided a greater stiffening effect in the composite. Behazin *et al*. also observed that biocarbon produced at high temperature had a much smaller pore size compared to biocarbon produced at low temperature. It is clear that pyrolysis conditions govern the properties of biocarbon which are integral to their performance as reinforcing fillers. Further, these attributes vary depending on the desired application and the nature of the matrix polymer.

## 2. Material and methods

Biocarbon was created by pyrolysis of soy hulls, light roast coffee chaff and dark roast coffee chaff at two temperatures and two time durations at each of the temperatures, producing a total of 12 unique biocarbons for characterization. In characterizing biocarbon, the factors of interest are the microstructure, including porosity and surface area; the elemental constitution; the functionality; and thermal and electrical conductivities [12,22,23].

### 2.1. Raw materials

Soy hulls were received from a local farm (Guelph, ON, Canada) as chips approximately 3 mm in diameter, then dried for 24 h at 85°C in an oven before pyrolysis. Both light roast coffee chaff roasted by Tim Horton's (Oakville, ON, Canada) and dark roast coffee chaff roasted by Club Coffee (Toronto, ON, Canada) were supplied by Competitive Green Technologies (Leamington, ON, Canada) and milled using a Retsch ZM 200 vortex mill to 1 mm then dried for 24 h at 85°C in an oven before pyrolysis.

### 2.2. Biocarbon preparation

**Figure 1.**
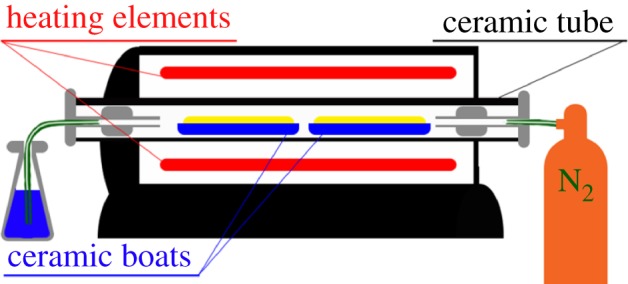
Schematic of horizontal tube furnace as used for the pyrolysis process.

Dry materials were weighed into ceramic boats and sealed into a Carbolite GHA 12/300 horizontal tube furnace with a model 3216 temperature controller (Carbolite Gero Ltd, UK), which was continuously purged with nitrogen at a flow rate of 1.9 l min^−1^. A schematic diagram depicting this set-up is shown in figure 1. The temperature of the furnace was ramped at a rate of 10°C min^−1^ to the holding temperature. The holding temperature was then sustained for the holding time (duration), before the furnace was allowed to cool at an average rate of approximately 1.5–2°C min^−1^. The pyrolysis holding temperatures used were 500 or 900°C, and the holding times were 15 or 30 min. After pyrolysis, the ceramic boats were weighed again to calculate yield. Before analysis, samples were ball milled for 1 h using a Fritsch Pulverisette 5 (Fritsch GmbH, Germany) with 1 cm zirconium dioxide balls and 500 ml zirconium dioxide jars with a biocarbon to ball mass ratio of 1 : 20. Samples were stored at 105°C for 24 h before analysis.

### 2.3. Scanning electron microscopy

Scanning electron microscopy (SEM) was used to estimate porosity and identify microstructure in raw or ground biocarbon. SEM was conducted in conjunction with energy-dispersive X-ray spectroscopy (EDS) using a Phenom ProX microscope (Phenom-World BV, The Netherlands). Micrographs were collected with an accelerating voltage of 10 kV, using the image mode, with images captured at a magnification of 10 000×. EDS was performed using an accelerating voltage of 15 kV on point mode at the same magnification.

### 2.4. Spectroscopic analysis

Fourier transform infrared (FTIR) analysis was conducted using a Nicolet 6700 spectrometer (Thermo Scientific, USA) in both attenuated total reflectance (ATR) and diffuse reflectance modes. Spectra were captured in ATR mode with 64 scans at a resolution of 4 cm^−1^ from 4000 to 500 cm^−1^. Spectra were captured in diffuse reflectance mode with 128 scans at a resolution of 4 cm^−1^ from 4000 to 500 cm^−1^. Diffuse reflectance mode samples were prepared with a 9 : 1 mix of potassium bromide and biocarbon powders.

Raman analysis was conducted using a Thermo Scientific DXR2 Raman microscope (Thermo Scientific, USA). Spectra were collected from several points across a powder sample of each investigated biocarbon. A 562 nm laser was used with a power of 1 mW through a 50× optical lens, with a 50 µm slit. Spectra were collected using six acquisitions of 5 s each. Spectral peaks were resolved for the wavenumbers between 900 and 1800 cm^−1^ using OMNIC software.

### 2.5. Thermogravimetric analysis and ash analysis

Thermogravimetric analysis (TGA) was performed under a nitrogen atmosphere, ramping the temperature at a rate of 10°C min^−1^ to a maximum of 900°C, and observing the change in mass of the sample over time. The mass ratios of certain components of either biomass or biocarbon can be identified by observing the temperature at which the components combust or evaporate as drops in the mass on a mass versus temperature graph. A TA Q500 (TA instruments, USA) was used for the TGA and for determining the ash content in accordance with ASTM E1131-08. Ash content was also determined through gravimetric methods burning the ash in a Lindberg Blue M baffle oven (Thermo Fisher Scientific, USA) in accordance with ASTM D1762-84.

### 2.6. Elemental analyses

Organic elemental analysis (OEA) was conducted with a Thermo FLASH2000 (Thermo Fisher Scientific, USA) organic elemental analyser using the CHNS and O reactors following the procedures from the OEA Cookbook software (Thermo Fisher Scientific, USA) for analysing coal (coke).

EDS was used to confirm the elemental composition determined in the OEA of the biocarbon and to identify inorganic elements. EDS was performed in conjunction with SEM with the Phenom ProX (Phenom-World BV, The Netherlands) using an accelerating voltage of 15 kV, and magnification of 10 000×.

### 2.7. Brunauer–Emmett–Teller surface area analysis

The size of the particle and surface area were determined through the Brunauer–Emmett–Teller (BET) method using absorbance of nitrogen. These properties are affected by the conditions of pyrolysis as well as the ball milling conditions used to pulverize the biocarbon. The BET analysis of this biocarbon was performed using the Autosorb-iQ (Quantachrome Instruments, USA). Samples of approximately 75 mg were measured in 6 mm bulb cells, outgassed at 300°C for 3 h. Measurements were made with nitrogen gas using liquid nitrogen as a coolant. The multi-point BET method used measured 20 points for adsorption and 20 points for desorption at varying relative pressures, and five points were selected from the linear portion of the volume to relative pressure curve for BET analysis. Calculation was assisted by ASiQwin 5 software for multi-point BET analysis software (Quantachrome Instruments, USA).

### 2.8. Electrical conductivity

The biocarbon powder conductivity was measured by placing the powder in a 1 cm diameter insulating tube between two aluminium pistons with 1 and 9 kg of force from the top piston on the powder. The resistance of the powder inside the tube was measured using impedance spectroscopy using the Autolab PGSTAT302N FRA potentiostatic module with the AUT85394 Differential Electrometer-Amplifier. NOVA 1.8.17 software for EIS using the FRA Impedance Potentiostatic program for electrical impedance spectroscopy (Metrohm Autolab B.V., The Netherlands) was used. The resistance was confirmed using an ohmmeter. The resistance was used to calculate the conductivity of the powder.

### 2.9. Thermal conductivity, diffusivity and specific heat

Thermal properties are important for use of the biocarbons in composites. The thermal properties were determined using the Thermtest Hot Disk TPS 500 (Thermtest Inc., Canada) using a Kapton tape sensor placed in the centre of a hollow steel cylinder capped with two steel pistons filled with at least 5 mm of biocarbon surrounding the sensor. For this test, 120 mW of heating for 80 s was used. The Hot Disk Thermal Constants Analyzer 7.0.14 software was used for the analysis (Thermtest Inc., Canada).

## 3. Results and discussion

### 3.1. Lignocellulosic component analysis

The Van Soest method [24] was used to determine the neutral detergent soluble fraction and the acid soluble fraction to calculate the amount of cellulose, hemicellulose and lignin in each sample. Soy hulls were found to have the largest relative cellulose component making up 42% on dry weight basis, and more hemicellulose than lignin, 15% and 6%, respectively. However, the lignin component was higher than that of most grasses used to create biocarbons [25,26]. These findings are consistent with those found in literature determined using the same method (42% cellulose, 18% hemicellulose, 2% lignin) [27]. Both coffee chaff materials were high in lignin; the dark roast contained 21% and the light roast contained 32%, with low amounts of cellulose. The dark roast contained 24% cellulose and the light roast contained 13% cellulose, and very low amounts of hemicellulose; the dark roast contained 11% and the light roast contained 12% hemicellulose. These findings are consistent with those found to literature determined using the same method (24% cellulose, 12% hemicellulose, 18% lignin) [16]. The substantial lignin component of these materials will create biocarbons higher in carbon than biocarbons generated from grasses or woodchips [14].

### 3.2. Thermogravimetric analysis

**Figure 2.**
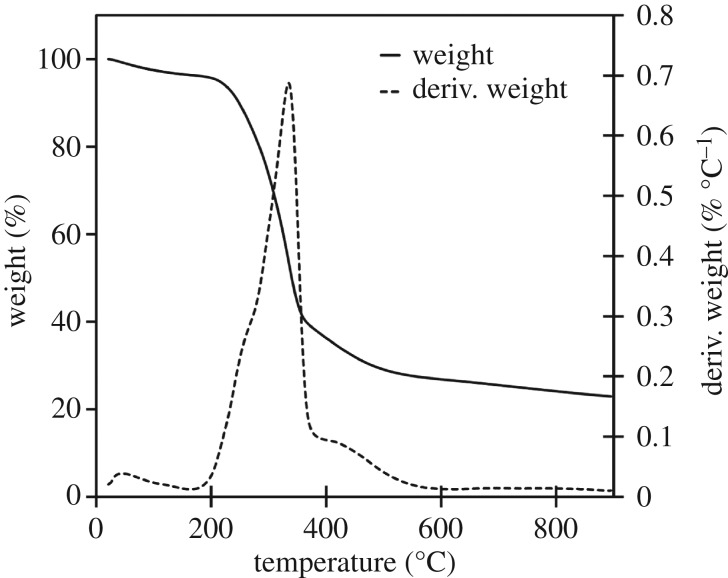
TGA and dTGA of raw soy hulls between 25 and 900°C, with hemicellulose, cellulose and lignin peaks visible in dTGA plot.

**Figure 3.**
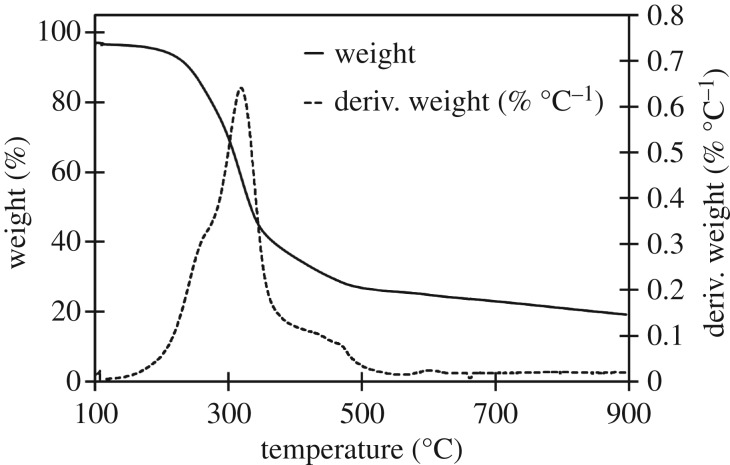
TGA and dTGA of raw dark roast coffee chaff between 100 and 900°C, with hemicellulose, cellulose and lignin peaks visible in dTGA plot.

**Figure 4.**
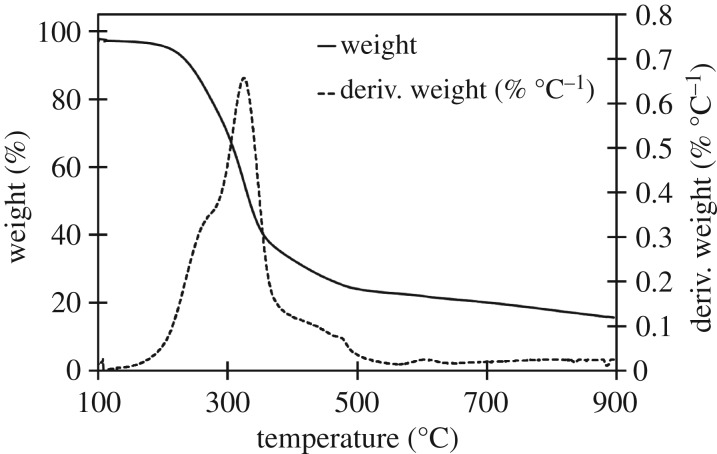
TGA and dTGA of raw light roast coffee chaff between 100 and 900°C, with hemicellulose, cellulose and lignin peaks visible in dTGA plot.

The three raw materials were analysed to identify the stages of decomposition and identify any characteristic temperatures of components which might not be decomposed in either high- or low-temperature biocarbons. Further analysis was done of each biocarbon assess thermal stability over the 100–900°C window. The derivative of the TGA with respect to temperature (dTGA) of the raw materials was graphed to improve resolution of small changes in mass, characteristic peaks in the dTGA chart were observed at 272, 331 and 377°C, as seen in the TGA and dTGA chart for soy hulls in figure 2. TGA and dTGA results for light and dark coffee chaff were like that of soy hulls as seen in the TGA and dTGA charts in figures 3 and 4. The peak at 272°C is associated with the decomposition of hemicellulose, the peak at 331°C is associated with the decomposition of cellulose and the peak at 377°C is associated with decomposition of lignin [28]. At 500°C, the low pyrolysis temperature in this study, lignin was the only lignocellulosic component not complete decomposed, this is common to all studied feedstocks. Stability is reached with the dTGA approaching zero before 900°C in all samples, this suggests that no unstable components remain in 900°C biocarbons.

**Figure 5.**
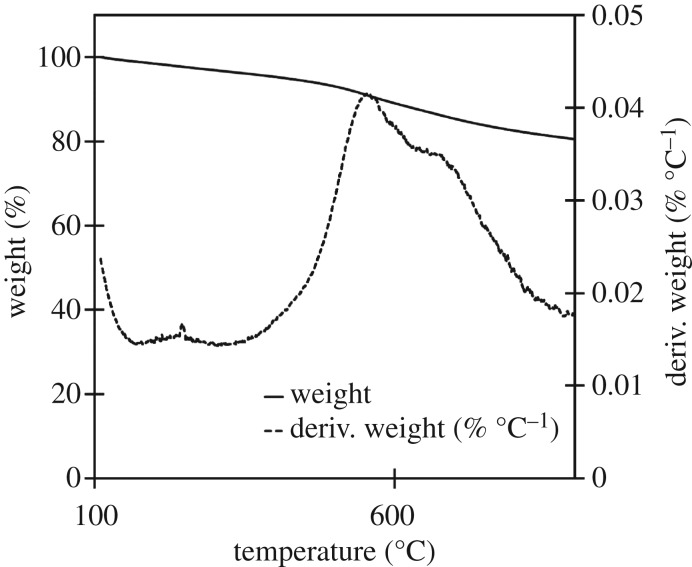
TGA of soy hull biocarbons pyrolysed at 500°C for 15 min displaying both the weight per cent remaining at a given temperature, and the derivative of the weight per cent remaining with respect to temperature.

**Figure 6.**
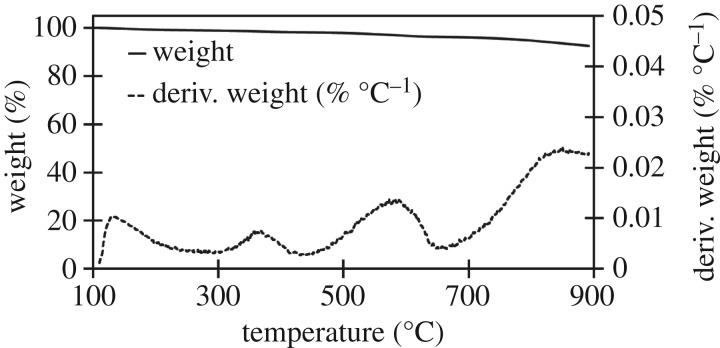
TGA of soy hull biocarbons pyrolysed at 900°C for 30 min displaying both the weight per cent remaining at a given temperature, and the derivative of the weight per cent remaining with respect to temperature.

**Figure 7.**
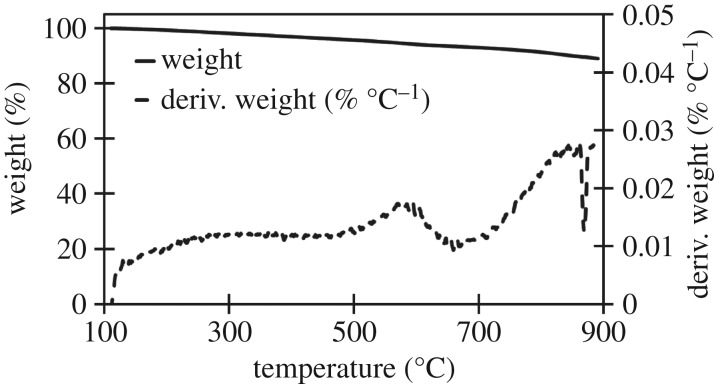
TGA of dark roast coffee chaff biocarbons pyrolysed at 900°C for 30 min displaying both the weight per cent remaining at a given temperature, and the derivative of the weight per cent remaining with respect to temperature.

**Figure 8.**
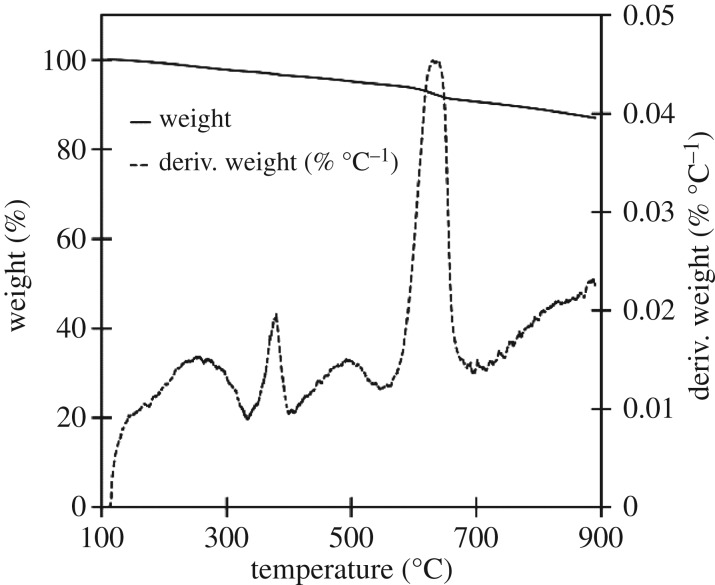
TGA of light roast coffee chaff biocarbons pyrolysed at 900**°**C for 30 min displaying both the weight per cent remaining at a given temperature, and the derivative of the weight per cent remaining with respect to temperature.

TGA of 500°C biocarbons revealed that thermal degradation began at 400°C and reached a maximum rate of decomposition at approximately 550°C at which point the remaining lignin was being decomposed; the chart of this analysis for soy hull biocarbon is shown in figure 5. TGA of 900°C soy hull biocarbons were thermally stable between 100 and 900°C with no prominent peaks appearing the dTGA analysis, as shown in figure 6. In both the coffee chaff samples, both (500°C and 900°C), significant degradation occurred at approximately 650°C; see figures 7 and 8. This may be related to the dehydrogenation of hydrocarbons at 672°C [29].

### 3.3. Yield and ash content

The biocarbon yield was measured on a dry weight basis comparing the mass of the raw feedstock before pyrolysis to the mass of resulting biocarbon. This yield decreased as the pyrolysis temperature increased and when the duration of pyrolysis increased. This result is consistent with the TGA of the raw materials seen in figure 2. This analysis revealed that the lignocellulosic biomass had not completely degraded at 500°C. The continued decomposition of biomass as the temperature is increased past 500°C causes the decrease in yield between low- and high-temperature biocarbons. Biocarbon yield is dependent on type of feedstock. Coffee chaff had a higher yield than soy hulls when pyrolysed at 500°C; this is likely to be due to the partial degradation from roasting coffee chaff. No difference in yield was observed for 900°C biocarbons. A summary of yields can be found in table 2.

**Table 1.** Abbreviations and pyrolysis parameters for biocarbon samples.

abbreviation	feedstock	temperature of pyrolysis (°C)	duration of pyrolysis (min)
DC1	dark roast coffee chaff	500	15
DC2	dark roast coffee chaff	500	30
DC3	dark roast coffee chaff	900	15
DC4	dark roast coffee chaff	900	30
LC1	light roast coffee chaff	500	15
LC2	light roast coffee chaff	500	30
LC3	light roast coffee chaff	900	15
LC4	light roast coffee chaff	900	30
SH1	soy hulls	500	15
SH2	soy hulls	500	30
SH3	soy hulls	900	15
SH4	soy hulls	900	30

**Table 2.** Yields of pyrolysis of soy hulls and coffee chaff at 500 and 900°C for 15 and 30 min, with standard deviations (see table 1 for abbreviations).

material	yield	ash content	
		ASTM E1131-08	ASTM D1762-84
SH1	30% (0.5769)	8.0% (1.0)	12.2% (0.08)
SH2	29% (0.4832)	8.1% (0.3)	12.6% (0.1)
SH3	26% (0.1751)	11.5% (1.5)	15.5% (0.2)
SH4	26% (0.2198)	11.0% (2.3)	16.5% (0.06)
LC1	33% (0.2732)	20.4% (2.8)	13.7% (0.4)
LC2	31% (0.2503)	12.4% (0.04)	13.7% (0.7)
LC3	26% (0.1681)	14.1% (0.6)	16.2% (0.7)
LC4	26% (0.1793)	15.5% (1.1)	18.8% (1.1)
DC1	33% (1.0829)	14.0% (1.3)	15.3% (0.7)
DC2	32% (0.3826)	12.9% (0.9)	15.6% (0.8)
DC3	27% (0.6924)	20.0% (0.08)	18.5% (0.5)
DC4	27% (0.4876)	15.1% (0.02)	18.5% (0.5)

Ash contents of industrial biocarbons are highly dependent on feedstock. For example, when fast growing, annual crops such as switchgrass or wheat straw are used, the resulting biocarbons have high ash contents ranging from 9 to 19% [9,12], while wood chip biocarbons typically have low ash contents ranging from 0.3 to 7% [9,29]. Ash contents in this study fell within the range of ash contents typically observed in annual grass crops (12–20%) [12]; detailed results of the ash content analysis can be found in table 2. The ash content of soy hull biocarbons was significantly lower than the coffee chaff biocarbons, and falls between the ash content of industrial wood chip biocarbon and grass crop biocarbon [12]. The coffee chaff biocarbons all had high ash contents, and the light coffee chaff was the highest. The high ash content of these materials is consistent with the high lignin content and the high metal content identified in the EDS analysis [30].

These trends in ash content are consistent between both methods of determination. The exact values of ash content obtained by each method are different for each sample. The ash content determined using ASTM E1131-08 was consistently 1–3% lower than the ash content determined using ASTM D1762-84 for each sample. This discrepancy is likely to be due to the continuous nitrogen purge of the furnace in the ASTM E1131-08 test carrying off a small amount of the fine biocarbon powder over the course of the experiment, and the smaller sample size used in ASTM E1131-08 reduces the chance of pockets of unreacted material forming. In addition to the difference in exact values, the ash contents determined by ASTM E1131-08 had higher standard deviations than those determined by ASTM D1762-84. Similar results have been observed when testing the ash content of identical biocarbons with different sample weights [31]. This imprecision may be due to the previously mentioned gas purge carrying away the highly mobile powder from the open crucible or the inhomogeneity of the biocarbon powder affecting the small, 10 mg sample more than the 1 g samples used in ASTM D1762-84. Owing to these inconsistencies caused by ASTM E1131-08, the results for LC1 and DC3 which are not consistent with the observed trend are considered outliers.

### 3.4. Fourier transform infrared spectroscopy

**Figure 9.**
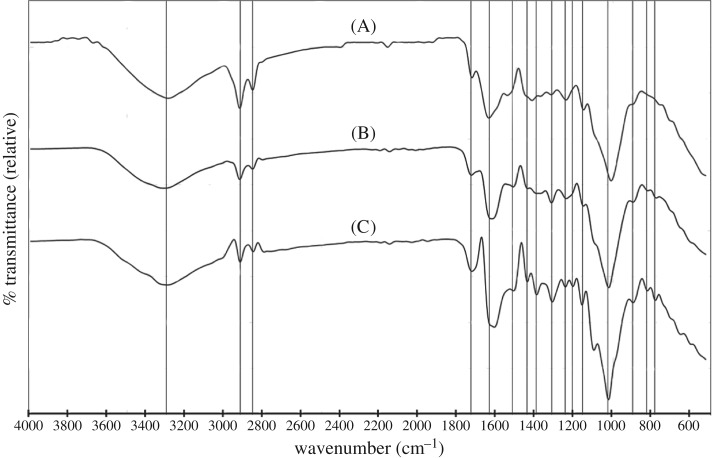
FTIR spectra of the raw soy hulls (A), light roast coffee chaff (B) and dark roast coffee chaff (C), ball milled for 4 h.

**Figure 10.**
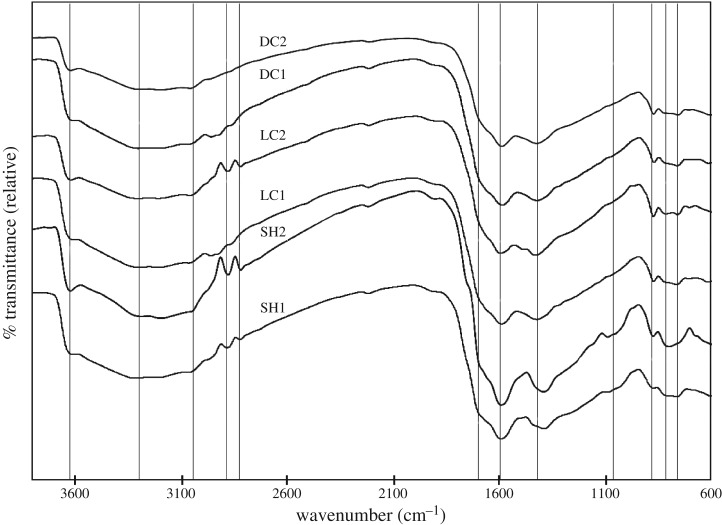
FTIR spectra of soy hull and coffee chaff biocarbon pyrolysed at 500 and 900**°**C for 15 and 30 min.

Soy hulls, light roast coffee chaff and dark roast coffee chaff raw materials were analysed using FTIR ATR spectroscopy, with prominent peaks, characteristic of specific functional groups and lignocellulosic components identified, as displayed in figure 9. The spectra of 500°C biocarbons of each of these feedstocks with pyrolysis durations of both 15 and 30 min were collected using FTIR diffuse reflectance spectroscopy, displayed in figure 10, and analysed noting the transformations in the prominent peaks identified in the raw biomass samples as well as prominent peaks which are emergent in the biocarbon. In each raw feedstock, prominent peaks indicated the presence of abundant hydroxyl groups, aliphatic carbon chains, carboxyl groups both conjugated and unconjugated with aromatic groups, aromatic groups, as well as two separate groups of peaks which together are characteristic of lignin, and cyclic carbohydrates, such as cellulose. In the biocarbon spectra, the hydroxyl group peak was less prominent, as were the peaks characteristic of aliphatic hydrocarbons, while the peaks characteristic of cyclic carbohydrates were absent. Peaks characteristic of aromatics, carboxyl groups and lignin were retained. Table 3 describes the peak assignments for the FTIR spectra analysed in this study.

**Table 3.** Significant peaks identified in IR analysis of raw soy hulls and coffee chaff and their derived biocarbons (see table 1 for abbreviations).

wavenumber	description	references
3320 − 3280 cm^−1^	hydroxyl group stretching	[32]
2915 + 2860 cm^−1^	asymmetric and symmetric C–H groups on aliphatic hydrocarbons	[32]
1770 + 1620 cm^−1^	carboxyl groups conjugated and unconjugated with aromatics	[33]
1513 + 1442 cm^−1^	aromatic alkene stretching	[33,34]
1350 − 1260 cm^−1^	in-plane bends of hydroxyl groups	[34]
1240 + 1215 cm^−1^	aromatic C–O stretching	[33]
1000 + 1100 cm^−1^	the ring and side group vibrations of backbone of any cyclic carbohydrates	[32]
818 + 775 cm^−1^	aromatic out-of-plane vibrations	[35]

Evidence of unique functionalities of raw feedstocks includes the different relative intensities of the peaks characteristic of aliphatic hydrocarbons and the difference in positioning of the peak characteristic of carboxyl groups (table 3). The aliphatic peaks were more pronounced in soy hulls than in light coffee chaff, and least pronounced in dark roast coffee chaff. The change in size and rightward shift in the 1639 cm^−1^ peak suggests that C=O bonds of the roasted materials were more frequently conjugated with aromatics. Common spectra for aromatic alkene stretching from lignin were identical for all samples.

The chemical transition from biomass to biocarbon is marked by the loss of functionality, the loss of carbohydrates and the retention of aromatics. The leftward shift and drop in intensity of the hydroxyl stretching peaks suggest that hydrogen bonds had been broken and most hydroxyl groups had decomposed completely [36]. The spectra indicating the presence of aliphatic hydrocarbons in the raw materials were nearly completely absent in the biocarbons; however, the peaks appeared faintly in the 15 min coffee chaff biocarbons. The peak that corresponds to carboxyl groups conjugated with aromatics was shifted to the left from its position in the raw material spectra. This was probably due to the complete dehydration of the sample and lack of secondary structure of the biocarbon [36]. Peaks characteristic of aromatic ring stretch in lignin appeared most prominently in the light coffee chaff biocarbon 30 min sample, which is consistent with the light coffee chaff being found to have the highest lignin content. The double peak at 2915 and 2860 cm^−1^, which corresponds with the C–C stretch in aromatics, appeared more stable in the soy hull biocarbon than in the coffee chaff biocarbon [36]. Some carbohydrates appeared to remain in these 500°C biocarbons as the peak that corresponds to C1 groups with B-glycosidic bonds still appeared, though it was at a lower relative transmittance. In addition, a minor peak at 1085 cm^−1^ was observed in the soy hull biocarbons, suggesting more cyclic carbohydrates remain intact after pyrolysis in this material over coffee chaff. This can be explained by the roasting treatment that the coffee chaff had been exposed to, which decomposed these molecules.

### 3.5. Scanning electron microscopy

**Figure 11.**
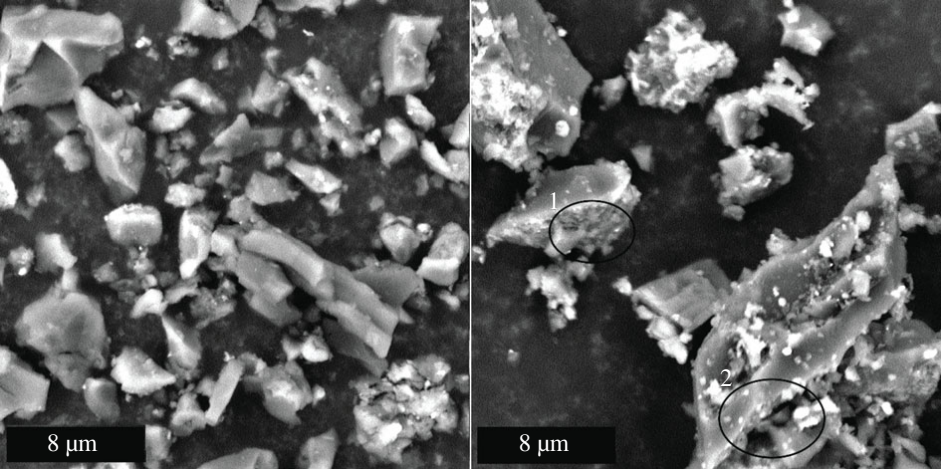
Biocarbon from dark roast coffee chaff 500°C 15 min (left) and 900°C 15 min (right). Phenom ProX SEM, accelerating voltage 10 kV, magnification 10 000×.

**Figure 12.**
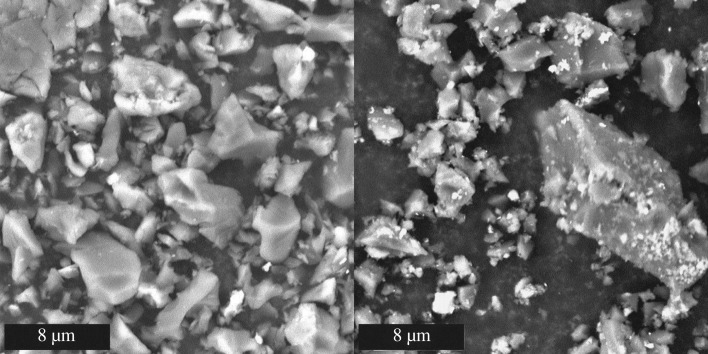
Biocarbon from soy hulls 500**°**C 15 min (left) and 900**°**C 15 min (right). Phenom ProX SEM, accelerating voltage 10 kV, magnification 10 000×.

Scanning electron microscope images of all 12 ball-milled biocarbons were captured to identify changes in microstructure with changing pyrolysis conditions and feedstocks. The micrographs showed irregular granular particles with smooth surfaces made up most of the 500°C biocarbon; in all biocarbon samples, smaller agglomerated globular particles existed. These two classes of particles can be distinguished in figure 11. In the coffee chaff biocarbons, particles were more agglomerated, while in soy hull biocarbons, they were more diffuse, figure 12. Through EDS analysis, it was established that these particles were higher in oxygen and metals than the smooth particles. The 900°C biocarbons formed a mix of larger and smaller irregular particles, the larger particles were rough. The roughened surface can be seen at point 1, circled in figure 11, and in the voids in the large particle at point 2 circled in figure 11. Among the smaller particles, both smooth and rough varieties were present. The light and dark roast coffee chaff biocarbons had fewer small irregular particles than the soy hull biocarbons.

The 900°C biocarbon particles show some porous structure, shown in figure 11 point 2, which corresponds to an increase in the surface area, causing the observed increase in electrical conductivity. This increase in porosity at high temperatures has been observed in biocarbon from other feedstocks [9], and is confirmed by the higher surface area observed in 900°C biocarbons.

### 3.6. Elemental analysis

Elemental analysis of all 12 biocarbons was conducted using both OEA and EDS analysis. The OEA served to accurately identify changes in key organic components; carbon, oxygen, nitrogen and hydrogen; corresponding to changes in feedstock, temperature of pyrolysis and duration of pyrolysis. EDS analysis was used to confirm the findings of this analysis, as well as identify which metals are most prevalent in the ash of these biocarbons. As temperature increases, functional molecules are gasified resulting in lower oxygen, hydrogen and nitrogen contents. Carbon to oxygen ratio provides a consistent comparison between samples and methods. Dark coffee chaff pyrolysed at 900°C had the highest C/O ratio, followed by light coffee chaff and soy hulls pyrolysed at 900°C which were not significantly different. The C/O ratio of the 500°C biocarbons from all feedstocks were not significantly different. Time of pyrolysis did not appear to have a significant effect on the elemental composition of biocarbon.

The carbon content by weight increased by 8.5% in dark roast coffee chaff, by 7.5% in soy hulls and by 9.3% in light roast coffee chaff.

The nitrogen content of the feedstocks ranged from 1 to 3% by weight with the N content falling as the pyrolysis temperature increased. This is higher than the nitrogen content of biocarbons from certain common feedstocks, as the N content of wood chips, rice hulls and rice straw-derived biocarbons falls between 0.3 and 0.8% [13,29]. The N content of the soy hulls and coffee chaff biocarbons was more similar to that of miscanthus or switchgrass biocarbons [12]. The higher nitrogen content suggests the presence of heterocyclic N, such as pyridines or pyrroles, and may increase the porosity of the high-temperature biocarbon [29].

The dehydrogenation of the biomass through pyrolysis at 500°C produced hydrogen contents of approximately 3%, which is similar to those found in the literature [12,13,29], and 900°C pyrolysis produced levels of approximately 0.5% which is much lower than the hydrogen contents in the other studies. This is likely to be due to the slower heating time and cooling time resulting in a longer dwell time at temperatures between 523 and 673°C, where most reactions resulting in dehydrogenation to CH_4_ and H_2_ occur [29].

### 3.7. Raman spectroscopy

In this study, Raman spectra were gathered from all 12 biocarbon species, derived from all three feedstocks, pyrolysed at both temperatures, and help for both holding times. Four Lorentzian peaks were fit in this range corresponding to the *G*, *G*_R_, *D* and *S* bands as identified in previous reports [37,38]. The following peak assignments were used: 1580 cm^−1^ for the *G* peak representing graphite *E*_2g_^2^, specifically aromatic ring quadrant breathing; 1360 cm^−1^ for the *D* peak caused by C–C bonds between aromatic rings in highly ordered aromatic structures with more than five rings [37,39]; 1550 cm^−1^ for the *G*_R_ peak caused by amorphous aromatic carbon structures with three to five rings; and 1210 cm^−1^ for the *S* peak caused by hydroaromatic rings and mixed aromatic *sp*^3^ bonded carbon structures [37]. The area of the *G* peak was compared to the area of the *D* peak to determine the ratio of graphite carbon to turbostratic and amorphous carbon [37,38].

**Figure 13.**
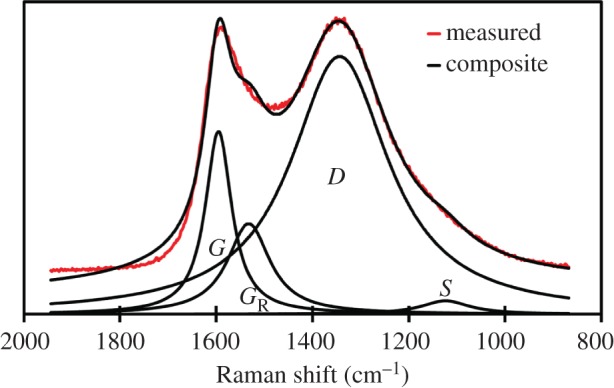
Resolution of Raman spectroscopy for light roast coffee chaff biocarbon pyrolysed at 900°C for 30 min.

These spectra were gathered to determine the *D*/*G* graphitic ratio for each biocarbon. The *D*/*G* ratio analysis requires resolution of the peak characteristic of carbon materials between Raman shifts of 800 and 1800 cm**^−^**^1^. An example of this four-peak resolution identifying each peak for the LC4 biocarbon is shown in figure 13. The resolved peaks were compared by calculating the ratio of the areas of the *D* and *G* peaks, to represent the ratio of graphitized carbon to un-graphitized carbon [37–40]. The area of the *G*_R_ peak was compared to the area of the *D* peak to model the ratio between small aromatics and large aromatics [38,40]. This ratio was used to understand what components of the low-temperature biocarbons are lost as the temperature of pyrolysis is increased.

The two most prominent peaks were the *D* and *G* peaks. The *D* peak typically found at a Raman shift of 1340 cm^−1^ corresponds to the disordered carbon to carbon bonds of a turbostratic structure, and the *G* peak at 1590 cm^−1^ corresponds to the ordered carbon to carbon bonds of graphite structures [38,40]. The *S* and *G*_R_ peaks were also present in all biocarbon samples, the *S*_L_ peak at 1200 cm^−1^ corresponds to carbon to carbon and carbon to oxygen bonds of oxygenated aromatic carbon structures, and *G*_R_ peak at 1540 cm^−1^ corresponds to amorphous carbon, and aromatics with three to five rings [37,38,40]. The *G*_R_/*D* area ratio did not change significantly with pyrolysis temperature or duration, as seen in table 4, which suggests that small aromatics are retained and the loss in mass is due to dehydrogenation, the decomposition of functional groups and gasification of aliphatic carbon molecules. As the time of pyrolysis increased, the *D*/*G* ratio declined in all biocarbons, as seen in table 4. In soy hull biocarbons, increased time of pyrolysis had a graphitizing effect, with the *D*/*G* ratio lower in 30 min biocarbons than 15 min biocarbons.

**Table 4.** Atomic content by element for each soy hull and coffee chaff biocarbon determined by OEA, with standard deviations (see table 1 for abbreviations).

material	elemental composition				Raman analysis	
	N	C	H	O	*D*/*G*	*G*_R_/*D* *10
SH1	2.45 (0.022)	67.18 (0.0038)	2.82 (0.027)	16.38 (0.16)	9.75 (1.133)	2.80 (0.150)
SH2	2.59 (0.086)	70.51 (1.86)	2.96 (0.11)	15.42 (0.14)	4.93 (0.232)	2.08 (0.0535)
SH3	1.94 (0.012)	74.51 (0.61)	0.76 (0.074)	9.65 (0.93)	4.10 (0.0480)	2.23 (0.0146)
SH4	1.83 (0.024)	74.44 (0.32)	0.55 (0.022)	9.05 (0.28)	2.31 (0.337)	3.01 (0.236)
LC1	3.01 (0.049)	65.11 (1.41)	2.94 (0.079)	14.49 (0.84)	14.49 (2.033)	3.13 (0.228)
LC2	3.04 (0.015)	65.62 (3.67)	2.74 (0.034)	12.24 (0.84)	4.66 (0.192)	2.09 (0.0442)
LC3	2.14 (0.19)	73.24 (1.63)	0.52 (0.076)	9.09 (0.52)	4.82 (0.192)	2.11 (0.0424)
LC4	1.84 (0.078)	71.69 (0.12)	0.48 (0.071)	10.39 (0.18)	5.01 (0.0832)	1.94 (0.0238)
DC1	2.81 (0.00046)	64.80 (1.17)	3.07 (0.040)	14.87 (1.29)	6.19 (0.759)	2.38 (0.112)
DC2	2.78 (0.032)	63.64 (0.52)	2.65 (0.057)	13.95 (1.29)	3.88 (2.279)	3.36 (0.0684)
DC3	2.05 (0.15)	72.53 (0.032)	0.55 (0.074)	7.64 (1.68)	4.50 (0.0849)	1.98 (0.0366)
DC4	1.85 (0.033)	67.19 (0.85)	0.73 (0.075)	11.40 (0.37)	4.93 (0.234)	2.08 (0.0535)

### 3.8. Brunauer–Emmett–Teller surface area analysis

The BET surface area of the soy hull biocarbon increased with the increase in the temperature of pyrolysis from 10.687 m^2^ g^−1^ in the 500°C 15 min to 12.872 m^2^ g^−1^ in the 900°C 30 min biocarbon. This is likely to be due to the further gasification at high temperatures. This supports the interpretation of SEM images in which roughness on the surface of 900°C biocarbons was evidence of greater porosity.

The type of feedstock had a larger effect on the surface area of biocarbon than the temperature of pyrolysis. The surface area of the 900°C 30 min biocarbon from light roast coffee chaff had a very low surface area of 3.465 m^2^ g^−1^. This may be due to the ash melting at high temperatures and filling the pores in the carbon as high-ash biocarbons have been observed to have particularly low surface areas [41–43]. The 500°C 15 min light roast coffee chaff biocarbon had a surface area of 11.743 m^2^ g^−1^. For this feedstock, lower temperature biocarbon is significantly lower in ash as shown in table 2, supporting the theory that ash content above a certain level is detrimental to surface area.

### 3.9. Thermal conductivity, diffusivity and specific heat

The thermal properties of the biocarbon were tested with a hot disc sensor placed in a column of powder. This was used to determine the thermal conductivity, diffusivity and specific heat of all 12 biocarbon powders. The thermal properties of each biocarbon powder are recorded in table 5. Of the 900°C biocarbons, soy hulls had the lowest conductivity and diffusivity; however, there is no significant difference in these properties among 500°C biocarbons. Thermal conductivities and diffusivities of 500°C hull waste biocarbons were lower than industrial biocarbons created at the same temperature from wood chips and perennial grasses; correspondingly, the specific heat of hull waste biocarbons was higher than the industrial reference [12]. The 900°C 15 min biocarbon was similar to these reference materials in all thermal properties. As the temperature of pyrolysis increased and time of pyrolysis increased, the specific heat of biocarbon decreased, the thermal conductivity increased and the thermal diffusivity decreased. In these respects, temperature was a stronger determining factor than time. Type of feedstock also influenced the thermal properties of the biocarbon, the soy hull biocarbons had the highest specific heat and dark coffee chaff had the lowest.

**Table 5.** Thermal properties including thermal conductivity, thermal diffusivity and specific heat of biocarbon samples, with standard deviations, as well as electrical conductivity of biocarbon samples, measured using impedance spectroscopy of a packed powder tube under 127 kPa of pressure with standard deviations (see table 1 for abbreviations).

material	thermal properties			electrical conductivity (mS m^−1^)
	conductivity (mW m^−1^ K^−1^)	diffusivity (10^−2^ mm^2^ s^−1^)	specific heat (MJ m^−3^ K^−1^)	
SH1	117 ± 4.67	3.36 ± 0.246	3.49 ± 0.259	22.7 ± 0.7215
SH2	119 ± 1.56	2.23 ± 1.34	3.66 ± 0.0980	13.9 ± 0.5346
SH3	163 ± 3.44	5.40 ± 0.0821	2.84 ± 0.0305	46.9 ± 0.3092
SH4	153 ± 0.411	5.01 ± 0.234	3.25 ± 0.0895	119 ± 4.297
LC1	119 ± 2.36	3.45 ± 0.134	3.60 ± 0.0585	8.40 ± 1.238
LC2	121 ± 7.85	4.34 ± 0.182	3.03 ± 0.0792	9.93 ± 1.027
LC3	171 ± 9.22	9.82 ± 0.440	2.59 ± 0.1324	5.78 ± 0.7176
LC4	202 ± 3.52	9.42 ± 0.214	2.16 ± 0.0568	3.50 ± 0.09596
DC1	118 ± 2.54	3.32 ± 0.0927	3.41 ± 0.0693	8.50 ± 1.139
DC2	132 ± 6.20	3.89 ± 0.209	3.03 ± 0.0177	8.89 ± 0.6218
DC3	196 ± 4.09	6.65 ± 0.696	2.00 ± 0.0536	307 ± 61.80
DC4	223 ± 6.88	12.38 ± 0.933	1.81 ± 0.0898	1.810 ± 16.25

These trends in specific heat are correlated with the ash content in the respective biocarbons. The specific heat of ash has a significantly lower specific heat than that of charcoal, it follows that the biocarbons which contain more ash have lower specific heats [44,45]. Past studies have also found that higher temperatures of pyrolysis can cause higher thermal conductivities, this can be explained by the increase in porosity at higher temperatures increasing the area of radiative heat transfer to the air [45]. However, this does not explain the higher thermal conductivities of the coffee chaff samples which have distinctly lower surface areas. The conductivity of the coffee chaff samples may be related to ash content as the thermal conductivity of ash is an order of magnitude greater than that of charcoals [46]. Given the relationship between the specific heat, thermal conductivity, thermal diffusivity and density, the increase in thermal diffusivity at higher pyrolysis temperature and longer pyrolysis durations can be expected as a reaction to the change in the other properties.

### 3.10. Electrical conductivity

The electrical conductivity for 500°C biocarbon was like that of 600°C industrial biocarbon (8–10 mS m^−1^) [12]. Soy hulls and dark coffee chaff 900°C biocarbons had higher conductivities, probably due to graphitization [42]; also, the higher temperature will further pit the surface by decomposing more organics. Light coffee chaff 900°C biocarbons showed lower conductivity, probably due to the high ash content [29]. Dark coffee chaff had significantly higher conductivity than most biocarbons; this is likely to be due to the higher carbon content. The electrical conductivities of the biocarbon powders are recorded in table 5.

Two major factors affecting electrical conductivity in biocarbon are graphite content and surface area [47]. In both soy hull and dark roast coffee chaff biocarbons, an increase in electrical conductivity is correlated with the increase in graphite content and increase in surface area observed as the duration and temperature of pyrolysis is increased. In light roast coffee chaff biocarbons, an increase in graphite content is observed; however, the high ash content probably lowers the surface area, negating the conductive effect of the graphite.

## 4. Conclusion

Coffee chaff and soy hull biocarbons have higher yields and ash contents than both wood chip and perennial grass crop biocarbons. As the temperature and duration of pyrolysis was increased, the ash content, surface area and carbon content of the biocarbon increased, affecting the thermal and electrical conductivities, specific heat graphite content, surface functionality and thermal stability. The increase in ash content, observed by two ASTM ash content determination methods, is correlated with an increase in the thermal conductivity and specific heat, and a decrease in electrical conductivity. The increase in surface area, as determined by BET surface area analysis, is correlated with an increase in both electrical and thermal conductivity. The increased carbonization of biocarbon, as determined by CHNS-O elemental analysis and energy dispersion spectroscopy, is correlated with increased graphitization determined by Raman spectroscopy, decreased surface functionality determined by infrared spectroscopy and increased thermal stability determined by TGA. The process of carbonization is more complete in coffee chaff because it has been partially carbonatized in the roasting process. Soy hulls are a suitable feedstock for most industrial applications of biocarbon including composite applications. Coffee chaff biocarbons are high in ash which is not ideal for composite or filtration applications; however, with further treatment to remove ash, the high-temperature dark roast coffee chaff could be used where electrical conductivity is a desirable property.

## Supplementary Material

ESM descriptions.docx

## Supplementary Material

RSOS ESM.zip

## Supplementary Material

RSOS ESM Raman.zip

## Data accessibility

The datasets generated during this research and used in this article have been uploaded as part of the electronic supplementary material.
