# Supplementary material for: Characterization of biocarbon generated by high- and low-temperature pyrolysis of soy hulls and coffee chaff: for polymer composite applications
Source: R Soc Open Sci. 2018 Aug 22;5(8):171970. doi: 10.1098/rsos.171970 (PMC6124110; doi:10.1098/rsos.171970)
Supplement: ESM descriptions.docx [file rsos171970supp1.docx]

**ESM descriptions**

1. **Ash content**

Ash content as determined by furnace method.

1. **Electrical and thermal properties.**

Resistance, conductivity and diffusivity values for each feedstock tested.

1. **Organic elemental analysis**

Results from organic elemental analysis of biocarbons.

1. **Van Soest fractions**

Values of lignin, cellulose and hemicellulose for each feedstock.

1. **Yield**

Raw values for calculation of yield for each biocarbon by feedstock.

1. **KBr FTIR**

FTIR files from tests done using potassium bromide stage.

1. **Raw FTIR**

FTIR files from tests done using ATR mode.

1. **TGA ash**

Ash content as determined by thermogravimetric method.

1. **Raman**

Raw files for Raman spectra of biocarbon.
